# Does high sugar intake really alter the oral microbiota?: A systematic review

**DOI:** 10.1002/cre2.640

**Published:** 2022-08-09

**Authors:** María del Pilar Angarita‐Díaz, Cristian Fong, Claudia M. Bedoya‐Correa, Claudia L. Cabrera‐Arango

**Affiliations:** ^1^ School of Dentistry, Universidad Cooperativa de Colombia Villavicencio Colombia; ^2^ School of Medicine, Universidad Cooperativa de Colombia Santa Marta Colombia; ^3^ School of Dentistry, Universidad Cooperativa de Colombia Medellín Colombia

**Keywords:** bacteria, dental caries, dysbiosis, microbiota, sugar

## Abstract

**Objectives:**

Diet is one of the main factors influencing the diversity and interactions of the oral microbiota. The purpose of this study is to determine the impact of sugar intake on the microbial diversity and bacteria that predominate under these conditions.

**Material and Methods:**

A systematic review was conducted according to the Preferred Reporting Items for Systematic Reviews and Meta‐Analyses (PRISMA) guide, using the PubMed, Scopus, and Science Direct databases and combinations of the words “microbiota,” “microbiology,” “bacteria,” “sugars,” “dysbiosis,” “caries,” “microbiome,” “oral microbial,” and “oral microbiota profile pattern.” The selection criteria included year, language, type of publication, comparison of microbiota during low and high sugar intake, and bacterial identification by molecular sequencing of the 16S subunit of ribosomal RNA.

**Results:**

Out of a total of 374 papers that came up after the initial search, 8 met the criteria for this review. The papers included research on populations comprising children, young adults, and adults, with most of the studies reporting selection criteria for the participants and using validated instruments to determine sugar intake. Apart from one study, all others reported for high sugar intake conditions a significant decrease in microbial diversity of the oral microbiome and the predominance of several bacterial genera or species, including *Streptococcus*, *Scardovia*, *Veillonella*, *Rothia*, *Actinomyces*, and *Lactobacillus*.

**Conclusions:**

Sugar‐rich diets have a significantly unfavorable effect on the diversity and balance of oral microbiota; however, further studies are required to determine the exact role of sugar in microbial interactions.

## 1 INTRODUCTION

The oral cavity has a great diversity of microorganisms whose presence depends on their interactions with several host factors, such as chemical and physical conditions of the oral cavity, immune response, nutritional habits, and oral hygiene, as well as interactions with other microorganisms (He et al., 2009). These interactions can occur via symbiotic processes, thereby favoring the balance or “eubiosis” of the oral ecosystem. They can also occur through the alteration of balance or “dysbiosis” of the oral microbiome, which causes changes in the number and type of microorganisms (Iebba et al., 2016).

The above is explained by an ecological hypothesis, which states that any unfavorable, intense, and persistent change in the host factors will trigger an imbalance (dysbiosis) of the oral microbiota and the ensuing predominance of disease‐causing microorganisms that adapt to the new environment. This hypothesis also explains the occurrence of caries. An increase in the intake of fermentable carbohydrates, such as sucrose, increases the number of bacteria whose metabolism produces acids that demineralize the tooth enamel, thereby leading to caries (Marsh, 2006; Nyvad & Takahashi, 2020).

In addition to the production of acids (acidogenicity), other virulence properties associated with cariogenic bacteria are their abilities of synthesizing more acids in a low‐pH medium (aciduricity), being resistant to this pH level without restricting their cellular functions (acidophilicity), and producing glucans and fructans, which promote their adherence to the dental surface and serve as a nutrient reserve (Dang et al., 2018; Kuramitsu & Wang, 2006). These properties adversely influence the prevalence and incidence of caries by hindering the oral immune response and the action of saliva as a buffer system (Rosier et al., 2018).

The advent of genetic and molecular analysis of oral diseases such as periodontitis or squamous cell cancer (Ferlazzo et al., 2017; Isola et al., 2022) has made it possible to explain the cellular mechanisms that cause them or the genetic factors that generate susceptibility to these conditions. The molecular studies of the oral microbiota of patients with caries have identified a complex composition of cariogenic microorganisms, which varies among patients, type of caries, lesion (initial or advanced), and location of caries, among other factors (Manzoor et al., 2021). This has led to the proposal of several prevention and control measures that no longer focus on only one or two types of microorganisms but on a group of microorganisms with common properties (Philip et al., 2018).

Different studies (ecological, epidemiological, experimental in vitro, etc.) have demonstrated a relationship between high sugary food consumption and the prevalence of caries. Thus, indicating an association with dysbiosis predicted by ecological theory (Manzoor et al., 2021; Moynihan & Kelly, 2014; Tinanoff & Palmer, 2000; Zhu et al., 1997). However, there is also a considerable number of studies that do not report the same relationship, probably owing to several factors, such as the variability in the patterns of sugar consumption, which further affects the exposure duration of teeth to sugar; dietary measurement instruments, which only provide an approximation of the actual sugar consumption; reporting time of sugar consumption patterns, which are annual, while the formation of caries can last several years, and factors influencing the prevalence of caries, such as mineral content in the diet (fluoride, calcium, and phosphorus), health care, oral hygiene habits, level of education (Touger‐Decker & van Loveren, 2003), and the presence of other diseases.

The role of sugar in caries has been associated with the establishment of environmental conditions that trigger dysbiosis and, therefore, the predominance of cariogenic bacteria. Therefore, the aim of this study is to determine the impact of high sugar intake on the oral microbial diversity and the types of microorganisms that predominate under these conditions. This will further contribute to the body of information regarding the relationship between diets rich in sugar and their effect on dental caries.

## 2 MATERIALS AND METHODS

### 2.1 Search strategy and study selection

A systematic review was conducted to determine the effect of sugar intake on oral microbiota diversity. The identification, screening, selection, and inclusion stages described in the Preferred Reporting Items for Systematic Reviews and Meta‐Analyses (PRISMA) guide (Liberati et al., 2009) were followed for searching and selecting the relevant papers from the databases.

The search was conducted between May and June 2021. The following keywords were used for the search: “microbiota” (MeSH), “microbiology” (MeSH), “bacteria” (MeSH), “sugars” (MeSH), “dysbiosis” (MeSH), “caries,” “microbiome,” “oral microbial,” “oral microbiota,” “profile,” and “pattern,” using different combinations and the Boolean operators “AND” and “OR” according to relevance (Table 1). Furthermore, we performed a manual search of the references cited in the selected papers.

**Table 1:** Search strategy designed for each database

Database	Search strategy	Results
Pubmed	(caries) AND (sugar) AND (microbiome OR microbiota OR microbiology OR bacteria) AND (dysbiosis)	23
	(“oral microbial” OR “oral microbiota” OR “oral microbiology”) AND (profile or pattern) AND (sugars)	27
Scopus	(caries) AND (sugar) AND (microbiome OR microbiota OR microbiology OR bacteria) AND (dysbiosis)	113
	(oral) and (microbial OR microbiota OR microbiology) AND (profile or Pattern) and (“sugars”)	52
Science Direct	(caries) AND (sugar) AND (microbiome OR microbiota OR microbiology OR bacteria) AND (dysbiosis)	150
	(oral) and (microbial OR microbiota OR microbiology) AND (profile or Pattern) AND (sugars)	124

The databases employed in this review were PubMed, Scopus, and Science Direct. We used the filters provided by the databases to select only the papers published from 2010 onward, in English and Spanish and as original studies (clinical trials, cohort studies, case–control studies). Reviews, meta‐analyses, gray literature, book chapters, degree projects, theses, abstracts, and participation in congresses, among other sources, were excluded.

Two researchers independently searched for the relevant papers (M. P. A.‐D. and C. J. F.) Once the relevant studies were extracted, the duplicates were eliminated. Then, four researchers (M. P. A.‐D., C. J. F., C. M. B.‐C., and C. L. C.‐A.) reviewed the titles and abstracts of the remaining papers and selected the studies (*κ* value > 0.80) that met the following inclusion criteria: (1) studies comparing the bacterial presence in patients with high or low sugar intake and/or frequency in some of the phases of the methodology; (2) patients of any age, sex, or origin; (3) full‐text studies; and (4) studies identifying bacteria through molecular sequencing of the 16S ribosomal RNA (rRNA) subunit. The exclusion criteria were as follows: (1) comparative studies evaluating variables other than sugar consumption; (2) studies carried out with patients with systemic diseases, under antibiotic treatment, or other types of drugs; (3) in vitro studies. Ultimately, the main investigator verified the final selection of the studies.

### 2.2 Data extraction process

Three researchers performed a qualitative synthesis of the variables for the papers included in the study (M. P. A.‐D., C. J. F., and C. M. B.‐C.), which comprised the authors and year of publication, type of study, patients, origin, selection criteria, method of determining sugar consumption, amplified region, relevant results, and bacteria identified in patients with high sugar intake.

### 2.3 Level of evidence and risk of bias

The quality of the papers included in the review was independently assessed by two researchers (M. P. A.‐D. and C. J. F.) using the Newcastle Ottawa scale (Stang, 2010). The parameters for assessing the risk of bias in the cohort studies included the selection of the cohort (the representativeness of the exposed cohort, selection of the unexposed cohort, determination of the exposure, and demonstration that the result of interest was not evident at the beginning of the study), comparability of the cohorts based on the design or analysis, and finally, the parameters for results (sufficiently long follow‐up period to produce results and the adequateness of the cohort follow‐up period).

Regarding the case–control and experimental studies, the parameters were the selection of the cases and controls (appropriate case definition, representativeness of cases, selection of controls, and definition of controls), comparability of cases and controls based on design or analysis, and exposure (exposure determination, same case–control verification method, and nonresponse rate).

## 3 RESULTS

### 3.1 Selection of studies

**Figure 1:**
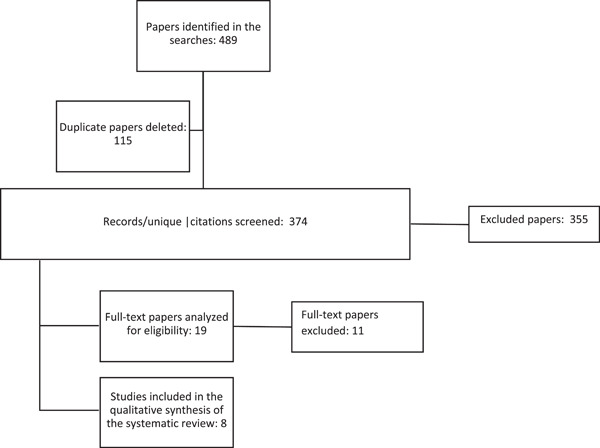
Information flowchart of the different stages of a systematic review

The systematic search of the three databases used in this study provided a total of 374 unique papers. After evaluating the titles and abstracts of all the papers, 19 were identified as being relevant to the objectives of this study. Consequently, these papers were read and analyzed in their entirety. However, only eight papers that met all the characteristics or criteria for this review were finally selected (Figure 1 and Appendix A).

### 3.2 Characteristics of the studies

The characteristics of the selected studies were that they were published between 2015 and 2021 and conducted in the following countries, China, Germany, Sweden, Denmark, and France. The study from Germany in 2020 included the same sample and results of the research published in 2018, along with comparisons with other variables associated with the diet (Table 2).

**Table 2:** Characteristics of the included studies

Author and year	Type of study	Origin/patients	Selection criteria	Method for determining sugar intake	Sample analyzed	Amplified region	Results	Bacteria identified in patients with high sugar intake
Tian et al. (2015)	Cohort study	Peking, China 24 children between 2 and 4 years old (36.54 ± 6.78 months). *No significant differences between the sexes. *Children who had received treatment for caries. *Two groups: With recurrence (*n* = 11); without recurrence (*n* = 3).	Systemically healthy children, no antibiotic consumption a month before sample collection, primary dentition, and more than 16 teeth and with at least seven decayed teeth and two or fewer filled teeth.	Sugar intake frequency questionnaire (before and 7 months after treatment).	Intact supragingival enamel biofilm before therapy and 1 and 7 months after therapy.	V3–V4	The group with the highest sugar consumption (recurrence group) showed lesser microbial richness and diversity and a significant decrease in *Lautropia mirabilis*.	Greater relative abundance and correlation with the frequency of sugar intake: *Campylobacter rectus*, *Capnocytophaga* sp. *HOT.323*, *Treponema* sp., *Selenomonas HOT.149*, *Prevotella HOT.313*, *Mitsuokella HOT.521*, *Veillonella atypica*, *Moryella HOT.419*, *Johnsonella ignava*.
Keller et al. (2017)	Case–control study	Copenhagen, Denmark 70 young adults, mainly women, healthy, and without obesity. *Two groups: (1) Sugar intake less than 5% (*n* = 30), mean age = 27 ± 8.8 years. (2) Sugar intake greater than 5% (*n* = 40), mean age = 30 ± 7.8 years. *Comparison between groups: No significant differences in terms of sex, age, or oral hygiene. Differences in caries experience being greater in the group with >5% sugar intake.	Varied diet with more than five or less than two intakes per day of foods with refined sugars for at least 3 months, without general and systemic diseases or medications that can alter the salivary flow, without recent or frequent treatment with antibiotics during the last year, without abundant dental plaque reflecting insufficient oral hygiene.	Instrument validated rapid Food Frequency Questionnaire (FFQ). The proportion of free sugars in relation to total energy was established with the help of the DANKOST program.	Supragingival biofilm was collected from the buccal and interdental surfaces of anterior and posterior teeth.	V3–V4	No significant differences were found in α‐diversity between the two groups.	Greatest significant prevalence: *Streptococcus sobrinus* and Prevotella melaninogenica. Higher prevalence, but without significant differences: *Streptococcus mutans*, *Scardovia wiggsiae*, *Rothia dentocariosa*, *Veillonella párvulai*, *Actinomyces* sp. *HOT.448*, and *Lactobacillus*.
Minty et al. (2018)	Case–control study	Toulouse, France 24 male patients, with an average age of 27.3 ± 4.7 years. Two groups: (1) Cases (*n* = 24): Rugby players with a significantly higher frequency of food intake (doubled) and snacks (more than doubled) and significantly higher values in the different indices (decay‐missing‐filled index (DMF), Significant Caries Index (SIC), and the plaque index). (2) Controls (*n* = 22): Nonrugby players, with a mean age of 26.6 ± 3.9 years.	No systemic diseases or salivary gland disorders, no use of antibiotics in the last 3 weeks before sample collection.	*Standardized record questionnaire to assess the amount and frequency of food intake and the type of food consumed (24‐h dietary recalls and dietary questionnaires).	Stimulated saliva.	V2–V4	The oral microbiota diversity was significantly lower in the rugby group, which had a higher frequency of food and snack intake.	Significantly higher relative abundance of *Streptococcus* and *Rothia*. Highest amount of *S. mutans*, *S. thermophilus*, and *S. sobrinus*, and a small increase in *S. gordonii*.
Anderson et al. (2018)	Interventional study	Freiburg, Germany 11 patients on a Western diet (45% carbohydrates), aged 21–56 years (mean 32 ± 12.7 years), with a similar sex ratio, mean COP of 8.1, and no patients with open carious lesions.	No severe chronic disease and no alteration in salivary glands or oral mucosa. No use of antibiotics or fluoride mouthwash for the past 30 days before sample collection, no dental treatment, no food intolerance or allergies, no eating disorders, and pregnancy or lactation.	Diet monitored through a validated FFQ. They also consumed 10 g of sugar during the day, distributed as 2 g five times a day between meals for 5 weeks.	Biofilm samples (collected in a splint with bovine enamel specimens). Collection in two phases: (1) Usual diet (for 5 days). (2) Usual diet along with consumption of 10 g per day (for 5 days).	V1–V2	*Significant decrease in oral microbiota species richness during sugar intake. Α diversity showed a decrease in 8 of the 11 study participants. *It was also determined that the microbial community before and during sugar intake was different (β‐diversity). *Significant decrease in the genera *Haemophilus* and *Aggregatibacter, Prevotella* and *Porphyromonas*.	*Significant differences in various taxa. Increase in phylum Firmicutes and genus *Streptococcus*. *Significant increase in S*. gordonii*, *S. parasanguinis*, and *S. sanguinis*.
Esberg et al. (2020)	Cohort study	Umeå Sweden 175 adolescents and young adults, between the ages of 17 and 21 years (mean: 18.1 years), a similar number of men and women, 4.4% are active smokers and 3.4% used to be smokers, 2.9% consume snuff and 8.6% previously did. 69.7% had caries and 31.1% had bleeding gums. The most consumed carbohydrates were starch (117 g/day), followed by sucrose (27 g/day), monosaccharides (19 g/day), and other sugars (23 g/day).	No antibiotic treatment (6 months before study), no systemic disease, no medication, and no communication in Swedish and English.	Validated instrument: FFQ.	Stimulated saliva.	V3–V4	α‐Diversity was significantly lower in the group with a high sugar intake, with differences within and between groups. β‐diversity also differed significantly between all the groups from the established groups in the analyses.	Abundant bacteria associated with high sugar intake: *Actinomyces*, *Bifidobacterium*, *Veillonella*, *Scardovia wiggsiae*, *S. mutans*, and *S. sobrinus*, among others. Another group with high sugar intake presented an increase in bacteria “not” characterized by being highly acidogenic, *Capnocytophaga*, *Leptotrichia*, *Prevotella*, and *Streptococcus*.
Anderson et al. (2020)	Interventional study	Freiburg, Germany 11 patients on a Western diet (45% carbohydrates), aged 21–56 years (mean 32 ± 12.7 years), similar sex ratio, mean COP of 8.1, no patient with open carious lesions.	No severe chronic disease, no alteration in salivary glands or oral mucosa, no use of antibiotics or fluoride mouthwash for the past 30 days before the study, no dental treatment, no food intolerance or allergies, no eating disorders, pregnancy or lactation.	Diet monitored through a validated FFQ. In addition, the participants consumed 10 g of sugar during the day, distributed as 2 g five times a day between meals for 5 weeks.	Biofilm samples (collected in a splint with bovine enamel specimens). Collection in two phases: (1) Usual diet (for 5 days). (2) Usual diet along with consumption of 10 g/day (for 5 days).	V1–V2	Species richness (α‐diversity) decreased significantly (*p* = .0027) with sucrose. The β‐diversity presented a significant difference between the usual diet and the diet with a high intake of sugar. A decrease in *Porphyromonas*, *Prevotella*, and *Granulicatella* was found. It was also determined that the oral microbiota differed significantly with respect to the other aspects of the diets (usual, yoghurt/milk and vegetable puree) during consuming the diet with a higher sugar content, where the richness of species decreased significantly (*p* = .008).	Main representative of the biofilm: *Streptococcus*.
Chen et al. (2021)	Cohort study	Bengbu, China 180 children with an average age of 11.3 ± 0.6 years (10.3–12.5 years), 102 female. Thirty children with high sugar intake (>1 serving/day), who had a higher prevalence of caries and gingivitis, most were boys (18/30).	Not stated in the paper.	Questionnaire for collecting information regarding the number of times participants consumed sugary drinks in the previous week.	Buccal swab was collected by swabbing the insides of both cheeks (last follow‐up).	V3–V4	Reduced oral microbiota diversity with high intake of sugar‐sweetened beverages. *Less abundance of *Haemophilus*, *Porphyromonas*, *Lautropia*, *Fusobacterium*, *Granulicatella*, *Capnocytophaga*, and *Alloprevotella*.	Greatest relative and significant (*p* < .05) abundance of *Streptococcus*, *Neisseria*, *Gemella*, *Veillonella*, *Leptotrichia*, *Rothia*, *Actinomyces*, *Prevotella*, *Corynebacterium*, and *Aggregatibacter*.
Esberg et al. (2021)	Cohort study	Umeå, Sweden 416 patients aged 16–79 years (average 29.6 years); 61.6% are female, 6.4% are/used to be smokers, 12.9% consume/used to consume snuff and 85.9% brush their teeth daily. Daily carbohydrate intake: 217 g Daily sucrose intake: 31 g.	No cognitive impairment, no serious illness, no recent antibiotic treatment, no difficulty in communicating in Swedish or English. Questionnaires with no more than 10% of the responses, or where the caloric intake, energy intake, height or weight was not credible, were excluded.	Validated instrument— rapid FFQ.	Stimulated saliva.	V3–V4	Ranking the participants by genus abundance yielded five clusters. The cluster with the highest sugar intake significantly had the lowest α‐diversity.	*Significantly increased genera: *Streptococcus*, *Lactobacillus*, *Saccharibacteria (TM7) [G−1]*, *Olsenella*, *Parascardovia*, *Rothia*, and *Scardovia* *Species with the greatest abundance: *S. mutans*, *S. gordonii*, *S. anginosus*, *S. intermedius*, and *Scardovia wiggsiae*.

Regarding the participants, the adults (aged 27–59 years) were predominant, followed by young adults (aged 19–26 years) and children (aged 6–11 years). Furthermore, three studies showed a higher proportion of females and in one study, the sample was comprised entirely of males. Regarding the selection criteria, most authors took into account that the participants were systemically healthy and that they had not used antibiotics for different periods (1–12 months) before sample collection. Exclusion criteria varied among studies, in some cases taking into account having undergone dental treatment, being intolerant to any food or suffering an eating disorder, being pregnant, lactating, and being obese. One study did not specify the selection criteria (Table 2).

In most studies, the strategy implemented to determine and compare the sugar intake of the participants was with the validated Food Frequency Questionnaire. On the other hand, two studies did not indicate whether the questionnaire was validated (Table 2).

The World Health Organization (WHO) considers a sugar consumption greater than 5% of the total daily energy intake as a high‐sugar diet to produce caries (WHO, 2015). Of the selected studies, only one used this threshold. Most studies defined the high and low sugar groups by calculating dietary sugar intake (E% or g/day) and/or the frequency of sweet foods consumed, or analyzing the group to which it corresponded based on the main objective of the research or by adding 10 g of sugar to the usual diet of the study participants (Table 2).

Among the samples collected and analyzed for microbial identification in the studies, the most used was stimulated saliva. The technique used in all the studies for bacterial identification was high‐throughput sequencing with the Illumina® MiSeq® system, where the V3–V4 region was mostly used for amplifying the ribosomal gene 16S (Table 2).

According to the clinical oral conditions, and in some cases, the plaque index or gingivitis prevalence of the participants, the caries experience of the participants was considered in the studies. In most studies, the participant groups with higher sugar intake showed a higher presence or prevalence of caries (Table 2).

### 3.3 Association of the microbial profile with sugar consumption

Most of the selected study results reported that the richness and diversity of oral microorganisms were significantly lowered in participants with higher sugar consumption, as observed from samples of either dental biofilm, saliva, or another type (Anderson et al., 2018, 2020; Chen et al., 2021; Esberg et al., 2020, 2021; Minty et al., 2018; Tian et al., 2015). This result was observed both in studies that compared two groups of participants with either high or low sugar intake or in samples taken from the same participants before and after a higher intake of sugar (Table 2). These same studies identified differences between the microbiome associated with low and high sugar consumption (β‐diversity).

The only study that did not report a significant effect of high sugar intake on the diversity of oral microbiota was the case–control study, wherein the threshold for defining high sugar intake was greater than 5% of the total daily caloric intake (Keller et al., 2017).

Another effect observed of high sugar consumption was an alteration in the abundance or prevalence of bacterial genera or species in the oral cavity. This change was associated with either the predominance of some bacteria or the decrease of others. A special case is the genus *Streptococcus*, the percentage of the abundance of this genus is negatively correlated with the diversity of the oral microbiota when high sugar consumption is present (Minty et al., 2018). The increase in species of the genus *Streptococcus* coincides with the decrease in the abundance of taxa such as *Proteobacteria*, *Pasteurellaceae*, *Bacteroidia*, and *Porphyromonas* (Anderson et al., 2018). In cases that demonstrated a significant increase in the bacterial population of the oral cavity under high sugar intake conditions, a greater abundance of the following bacteria was observed in the dental biofilm: *Campylobacter rectus*, *Capnocytophaga* sp. *HOT.323*, *Johnsonella ignava*, *Mitsuokella HOT.521*, *Moryella HOT.419*, *Prevotella melaninogenica*, *Prevotella HOT.313*, *Selenomonas_HOT.149*, *Streptococcus sobrinus*, *S. gordonii*, *S. parasanguinis*, *S. sanguinis*, *Treponema* sp., and *Veillonella atypica* (Anderson et al., 2018; Keller et al., 2017; Tian et al., 2015). In the case of saliva, *Actinomyces*, *Bifidobacterium*, *Capnocytophaga*, *Lactobacillus*, *Leptotrichia*, *Olsenella*, *Parascardovia*, *Prevotella*, *Rothia*, *Saccharibacteria (TM7) [G−1]*, *Scardovia*, *Scardovia wiggsiae*, *Streptococcus*, *S. mutans*, *S. thermophilus*, *S. sobrinus*, *S. gordonii*, *S. anginosus*, *S. intermedius*, and *Veillonella* were observed (Esberg et al., 2020, 2021; Minty et al., 2018). Finally, in the mouth‐swab samples, *Actinomyces*, *Aggregatibacter*, *Corynebacterium*, *Gemella*, *Leptotrichia*, *Neisseria*, *Prevotella*, *Rothia*, *Streptococcus*, and *Veillonella* were the most abundant (Chen et al., 2021).

On the other hand, bacteria such as *Aggregatibacter*, *Haemophilus*, *Lautropia mirabilis*, *Porphyromonas*, *Prevotella*, and *Granulicatella* were reported to be decreased in biofilm samples, and *Alloprevotella*, *Capnocytophaga*, *Granulicatella*, *Fusobacterium*, *Haemophilus*, *Lautropia*, and *Porphyromonas* were reported to be diminished in the buccal swab samples.

The genus *Streptococcus* was the most commonly reported among the most abundant or prevalent bacteria where the commonly (although not always significantly) identified species were *S. mutans* (*n* = 4), *S. sobrinus* (*n* = 3), and *S. gordonii* (*n* = 3). Other common bacteria species reported as having a higher abundance or prevalence were *Scardovia wiggsiae* (*n* = 3), as well as the genera *Veillonella* (*n* = 2), *Rothia* (*n* = 2), *Actinomyces* (*n* = 2), and *Lactobacillus* (*n* = 2) (Table 2).

### 3.4 Functional differences

The differences between microbiomes associated with high and low sugar consumption are not limited to changes in species abundance, functionally these communities showed differences. Most notably, the communities associated with high sugar intakes showed enrichment of sucrose and starch metabolism pathways (Esberg et al., 2020). These microbiomes also showed an increase in pathways associated with amino acid biosynthesis and metabolisms, protein export, CoA biosynthesis, membrane transport, signaling and cellular processes, and signal transduction (Chen et al., 2021; Esberg et al., 2021).

### 3.5 Level of evidence

All the selected studies had a low risk of bias since they met seven/eight of the nine characteristics according to the Newcastle Ottawa scale. Two of the cohort studies fulfilled eight of the items and another two fulfilled seven items. Regarding the case–control and experimental studies, most (*n* = 3) complied with eight and only one complied with seven of the nine items. Most of the studies failed to have a representative sample of the population (Appendix B).

## 4 DISCUSSION

In this review, it was found that the consumption of a sugar‐rich diet results in dysbiosis or imbalance of the oral ecosystem, and a functional modification of the microbiome, since most of the included studies reported a high‐sugar diet to have a significant effect on the diversity of oral microbiota, decreasing the population of some genera and species and increasing the predominance of others. These results were observed in studies where healthy participants were compared with individuals having caries (Hao et al., 2015; Hurley et al., 2019; Li et al., 2007).

The only study that did not find a significant decrease in oral microbial diversity as a response to a high‐sugar diet (Keller et al., 2017) used the WHO's suggestion of not exceeding 5% of the total daily energy intake (WHO, 2015). This can probably be explained by the fact that the concentration and/or frequency of sugar intake of some of the study participants were not high enough to impact their oral microbial diversity. Although the authors have not specified the average sugar intake of the participants who exceeded the threshold, they have discussed the need for further research with participants who show higher and more frequent consumption of refined sugars (Keller et al., 2017). This is essential, considering that there have been several studies that have reported that caries is more highly related to the frequency of sugar intake rather than the amount of sugar consumed (Anderson et al., 2009; Hong et al., 2018).

This review observed that high‐sugar diets have a significant impact on the oral microbiota. Among the most abundant and prevalent bacteria was the genus *Streptococcus*, since it is an important colonizer of the oral cavity, especially the teeth, due to the multiple adhesins that allow it to bind to the enamel surface and its ability to produce extracellular polysaccharides that enhance this adherence (Abranches et al., 2018). The species of this genus have a great capacity for using sugars and promoting oral microbiota dysbiosis through the production of acids, thereby leading to the acidification of the oral cavity and the adaptation of the bacteria to new environmental conditions (Abranches et al., 2018). *Streptococcus mutans* belongs to this genus and is the bacteria most studied and associated with dental caries because of its ability to metabolize a wide variety of carbohydrates, its multiple pathways of catabolizing sucrose, its various glycosyltransferase enzymes that promote the accumulation of biofilms and its great capacity of tolerating high concentrations of acids (Ravikumar et al., 2021). However, this bacteria is not always detected in individuals with caries, or it is less frequently seen compared with other bacteria (Simón‐Soro & Mira, 2015).

*Streptococcus sobrinus* and *S. gordonii* were the other streptococcal species which were most frequently mentioned in the different studies. *S. sobrinus* and *S. mutans* are the primary etiological agents of caries because they engender a greater impact on the incidence and experience of caries when detected together (Gross et al., 2012). Furthermore, *S. gordonii* has not been determined as a cause of caries, but on the contrary, as a protective agent against it due to its capacity to produce large amounts of alkali and its antagonism against *S. mutans* (Abranches et al., 2018). A study conducted with laboratory rats found that *S. gordonii* can survive in the teeth during high sugar intake (Tanzer et al., 2008), which could explain its presence in participants who consumed a sugar‐rich diet. This may also indicate the resilience of some bacteria against the changes in the host's biological conditions (Tanzer et al., 2008).

Other bacteria that were found to have high prevalence or abundance in the selected studies include *Scardovia wiggsiae*, *Veillonella*, *Rothia*, *Actinomyces*, and *Lactobacillus*. *Scardovia wiggsiae* has been considered a cariogenic bacteria due to its high levels found in children and adults with caries and its capacity to metabolize glucose and produce acetic acid via fermentation (McDaniel et al., 2021; Prabhu Matondkar et al., 2020). Furthermore, these bacteria are aciduric and have a significant tolerance toward fluoride that even exceeds that of *S. mutans* (Kameda et al., 2020). *Veillonella*, another one of the most prevalent genera in the human oral cavity (Mager et al., 2003), has been associated with either caries (Jagathrakshakan et al., 2015; Xu et al., 2018) or health conditions. This depends on the species since some species convert lactate into less acidic acids (Arif et al., 2008). Moreover, *Veillonella* is one of the genera stimulated by sucrose consumption (Minah et al., 1981), although a more recent study found a lower relative abundance of *Veillonella* in biofilms treated with sucrose compared with the control (Du et al., 2020).

The genus *Rothia* has species associated with caries because of its prevalence in the saliva and dental biofilms of individuals with caries (Jagathrakshakan et al., 2015). However, it has also been reported that the abundance of this genus caries‐free children (Xu et al., 2018). *Lactobacillus* is associated with caries progression and severity, and a higher decay‐missing‐filled (DMF) index in children indicates a higher count of this bacterium in saliva (Lapirattanakul et al., 2020). The genus *Actinomyces* has been associated with the pathogenesis of dental caries because it has been observed that the higher the rate of DMF in an individual, the greater the presence of this bacteria along with *S. mutans* and *Lactobacillus*. These bacteria have also been identified as a major pathogen of childhood root caries (Tang et al., 2003).

The functional differences found between microbiomes associated with low and high sugar consumption may signify adaptations of the microbiome to environmental changes (enrichment of acidogenic and acid‐tolerant species). This adaptation also involves mechanisms to counteract the pH reduction resulting from sugar consumption or the shift to anaerobic metabolism (Lommi et al., 2022; Wang et al., 2019). The activation of pathways associated with sugar metabolism indicates high metabolic activity (Chen et al., 2021).

In the study of oral diseases, it has been possible to identify possible biomarkers for disease or disease severity (Ferlazzo et al., 2017; Isola et al., 2022). It is possible that genera such as *Streptococcus, Veillonella*, *Rothia*, *Actinomyces*, and *Lactobacillus* that have been associated with caries may serve as biomarkers for this disease; in fact, the use of microbiome richness and abundance data has allowed the development of predictive models that have been able to differentiate patients with caries from healthy ones (Havsed et al., 2021; Li et al., 2021). Wang et al. (2019) used a classifier designed from defined microbiomes for caries patients. This classifier showed good performance in identifying caries patients (area under the curve of 98.53%) (Wang et al., 2019). Another aspect that supports this idea is that the composition of the microbiome between caries and healthy patients differs, the same has been observed for the microbiome associated with periodontitis (Shi et al., 2021). However, this requires further study due to interpersonal differences in the caries‐associated microbiome produced by age or diet.

The differences in the association of some bacteria with varying health conditions or diseases in the different studies are probably due to the type of sample, age range, or technique used, indicating the need for more studies to accurately determine the relationship between these bacteria and caries. It is important to note one of the studies where the authors found a conglomerate of bacteria characterized by “not” being associated with the carbohydrate metabolic pathway and being neither acidogenic nor aciduric in the saliva of participants with high sugar intake (Esberg et al., 2020). As the authors discussed, this may be due to other host factors, such as immunity and buffer capacity, which affects microbiome regulation, or due to an error in reporting the sugar intake of the study group (Esberg et al., 2020; Lamont et al., 2018; Rosier et al., 2018).

Although there is literature that does not report a significant relationship between caries and sugar consumption, most of the studies analyzed in this review clearly found an effect of high‐sugar diets on the prevalence of caries or other changes in the oral health of systemically healthy participants of different ages, sex, and origin (Anderson et al., 2018, 2020; Chen et al., 2021; Esberg et al., 2020; Tian et al., 2015).

It is important to specify that we could not perform an in‐depth comparison of the study results because of the heterogeneity of the samples of the selected studies (population, age ranges, sex, sample, and amplification region), which comprises one of the limitations of the study. However, the study results explain the impact of a high‐sugar diet on the composition of the oral microbiota. Furthermore, it should be noted that there are confounding factors, such as genetic heterogeneity and phenotypic diversity, within microbial species that make it more difficult to correlate the relationship between an event and a specific microbiota (Abranches et al., 2018). This has been observed in studies of phenotypic diversity of the *Streptococcus* strains of the same species where variations have been detected between the isolates that are directly related to the diseased or healthy biofilms (Abranches et al., 2018). Therefore, authors such as Abranches et al. (2018), suggest conducting studies where the community profiles of 16S rRNA are combined with other techniques, such as transcriptomics, proteomics, and metabolomics, to better understand the actual contribution of oral bacteria to health or disease (Abranches et al., 2018).

Another limitation of this study is that case–control studies and interventional studies were included. Therefore, it is important to conduct more studies, especially cohort studies, with adequate follow‐ups to help understand the effect of a sugar‐rich diet on the oral microbiota within the great diversity and factors that exist.

## 5 CONCLUSION

High sugar intake alters bacterial diversity of the oral cavity, causing the decline of some bacteria and enhancing the predominance of certain genera, such as *Streptococcus*, *Scardovia*, *Veillonella*, *Rothia*, *Actinomyces*, and *Lactobacillus*, which could serve as biomarkers for caries risk. However, the most frequently mentioned bacteria in the selected studies are not always considered causative agents of dental caries. Sugar consumption not only changes the makeup of the microbiome but also forces the microbiome to adapt, which increases carbohydrate metabolism and overall metabolic activity. Therefore, this review suggests conducting further studies that include techniques that allow for determining the true contribution of sugar in the predominance of bacteria and the interactions between such bacteria and the host factors.

## AUTHOR CONTRIBUTIONS

*Conceptualization, methodology, data extraction, drafted the manuscript*: María del Pilar Angarita‐Díaz, Cristian Fong, and Claudia M. Bedoya‐Correa. *Data extraction and reference organization*: Claudia L. Cabrera‐Arango.

## CONFLICT OF INTEREST

The authors declare no conflict of interest.

## Data Availability

All relevant data are within the manuscript.

## APPENDIX A

(Table A1).

**Table A1:** Excluded papers and criteria for their exclusion

Papers	Exclusion criteria
Rudney, J. D., Jagtap, P. D., Reilly, C. S., Chen, R., Markowski, T. W., Higgins, L., Johnson, J. E., & Griffin, T. J. (2015). Protein relative abundance patterns associated with sucrose‐induced dysbiosis are conserved across taxonomically diverse oral microcosm biofilm models of dental caries. *Microbiome, 3*, 69.	1. The study was carried out with a biofilm model of dental caries.
Goodson, J. M., Hartman, M. L., Shi, P., Hasturk, H., Yaskell, T., Vargas, J., Song, X., Cugini, M., Barake, R., Alsmadi, O., Al‐Mutawa, S., Ariga, J., Soparkar, P., Behbehani, J., & Behbehani, K. (2017). The salivary microbiome is altered in the presence of a high salivary glucose concentration. *PLoS One, 12*(3), e0170437.	1. Patients with diabetes. 2. The bacterial identification technique was not 16S ribosomal RNA (rRNA) subunit sequencing (DNA probes).
Xu, H., Tian, J., Hao, W., Zhang, Q., Zhou, Q., Shi, W., Qin, M., He, X., & Chen, F. (2018). Oral microbiome shifts from caries‐free to caries‐affected status in 3‐year‐old Chinese children: A longitudinal study. *Frontiers in Microbiology, 9*, 2009.	1. The objective focused on investigating the dynamics of supragingival microbiota change in 3‐year‐old children who developed caries in a 12‐month period or remained healthy. 2. The groups compared (healthy or with caries) showed no differences in the frequency of intake of sugary foods and beverages.
Jurczak, A., Jamka‐Kasprzyk, M., Bębenek, Z., Staszczyk, M., Jagielski, P., Kościelniak, D., Gregorczyk‐Maga, I., Kołodziej, I., Kępisty, M., Kukurba‐Setkowicz, M., Bryll, A., & Krzyściak, W. (2020). Differences in sweet taste perception and its association with the *Streptococcus mutans* cariogenic profile in preschool children with caries. *Nutrients, 12*(9), 2592.	1. The objective was not to identify differences at the microbiome level but rather the perception of sweet taste. 2. The bacterial identification technique was not 16S rRNA subunit sequencing (DNA probes).
Indiani, C. M. D. S. P., Rizzardi, K. F., Crescente, C. L., Steiner‐Oliveira, C., Nobre‐Dos‐Santos, M., & Parisotto, T. M. (2020). Relationship between mutans Streptococci and Lactobacilli in the oral cavity and intestine of obese and eutrophic children with early childhood caries—preliminary findings of a cross‐sectional study. *Frontiers in Pediatrics, 8*, 588965.	1. Obese children 2. The objective focused on determining the quantification of *Streptococcus mutans* and Lactobacilli in four groups of participants. 3. The bacterial identification technique was not 16S rRNA subunit sequencing (DNA probes).
Kalpana, B., Prabhu, P., Bhat, A. H., Senthilkumar, A., Arun, R. P., Asokan, S., Gunthe, S. S., & Verma, R. S. (2020). Bacterial diversity and functional analysis of severe early childhood caries and recurrence in India. *Scientific Reports, 10*(1), 21248.	1. Objective focused on the diversity of the bacteriome of the teeth in children with caries‐free (CF), severe early childhood caries (SC), and recurrent caries (RC). 2. They did not compare the bacteriome according to the sugar intake.
Burcham, Z. M., Garneau, N. L., Comstock, S. S., Tucker, R. M., Knight, R., & Metcalf, J. L. (2020). Genetics of taste lab citizen scientists. Patterns of oral microbiota diversity in adults and children: A crowdsourced population study. *Scientific Reports, 10*(1), 2133.	1. The only exclusion criterion was age. 2. The main objective focused on comparing the microbiota between adults and children. 3. Researchers do not report the microbiota of participants with high and low consumption of sugar and sugar‐free beverages.
Bostanci, N., Krog, M. C., Hugerth, L. W., Bashir, Z., Fransson, E., Boulund, F., Belibasakis, G. N., Wannerberger, K., Engstrand, L., Nielsen, H. S., & Schuppe‐Koistinen, I. (2021). Dysbiosis of the human oral microbiome during the menstrual cycle and vulnerability to the external exposures of smoking and dietary sugar. *Frontiers in Cellular and Infection Microbiology, 11*, 625229.	1. The comparative groups do not fit the objectives of the review (group that did not use any hormonal contraceptives, Group 2 that used combined oral contraception). 2. The groups did not show variations in sugar consumption. (sugar consumption did not vary throughout the menstrual cycle or between the contraceptive groups).
Manzoor, M., Lommi, S., Furuholm, J., Sarkkola, C., Engberg, E., Raju, S., & Viljakainen, H. High abundance of sugar metabolisers in saliva of children with caries. *Scientific Report, 11*(1), 4424.	1. The objective of the study was the difference in microbiota between children with and without caries regardless of sugar consumption between groups.
Grier, A., Myers, J. A., O'Connor, T. G., Quivey, R. G., Gill, S. R., & Kopycka‐Kedzierawski, D. T. (2021). Oral microbiota composition predicts early childhood caries onset. *Journal of Dental Research, 100*(6), 599–607.	1. Objective focused on identifying the composition of the microbiota between children with early caries and healthy children. 2. Sugar consumption was not measured.
Zheng, H., Xie, T., Li, S., Qiao, X., Lu, Y., & Feng, Y. (2021). Analysis of oral microbial dysbiosis associated with early childhood caries. *BMC Oral Health, 21*(1), 181.	1. Objective focused on identifying the composition of the microbiota between children with early caries and healthy children. 2. Sugar consumption was not measured.

## APPENDIX B

See (Tables B1, B2).

**Table B1:** Cohort studies were selected for the systematic review

Item	Selection part				Comparability part		Outcome part			Total
	Item 1	Item 2	Item 3	Item 4	Item 5	Item 6	Item 7	Item 8	Item 9	
Tian et al. (2015)		*		*	*	*	*	*	*	7
Esberg et al. (2020)		*	*	*	*	*	*		*	7
Chen et al. (2021)	*	*		*	*	*	*	*	*	8
Esberg et al. (2021)	*	*	*	*	*	*	*		*	8

*Note*: Items: 1, representativeness of the exposed cohort; 2, selection of the unexposed cohort; 3, determination of exposure; 4, demonstration that the result of interest was not present at the beginning of the study; 5 and 6, cohort comparability based on design or analysis; 7, outcome assessment; 8, follow‐up that is long enough to produce results; 9, adequacy of the cohort follow‐up period.

**Table B2:** Case–control and intervention studies were selected for systematic review

Item	Selection part				Comparability part		Exposure			Total
	Item 1	Item 2	Item 3	Item 4	Item 5	Item 6	Item 7	Item 8	Item 9	
Keller et al. (2017)	*		*	*	*	*	*	*	*	8
Minty et al. (2018)	*			*	*	*	*	*	*	7
Anderson et al. (2018)	*		*	*	*	*	*	*	*	8
Anderson et al. (2020)	*		*	*	*	*	*	*	*	8

*Note*: Items: 1, adequate case definition; 2, representativeness of cases; 3, selection of controls; 4, definition of controls; 5 and 6, comparability of cases and controls based on design or analysis; 7, determination of exposure; 8, same method of verification of cases and controls; 9, nonresponse rate.
